# Effect of Controlled Hypotension on the Outcomes of Stapes Surgery

**DOI:** 10.1055/s-0045-1811642

**Published:** 2025-10-09

**Authors:** António Andrade, João Viana Pinto, Pedro Marques, Manuel Mendes Leal, Carla Pinto Moura

**Affiliations:** 1 Department of Otorhinolaryngology, Centro Hospitalar Universitário São João, Porto, Portugal; 2 Department of Surgery and Physiology, Faculdade de Medicina da Universidade do Porto, Porto, Portugal; 3 Centre for Health Technology and Services Research, Faculdade de Medicina da Universidade do Porto, Porto, Portugal; 4 Department of Genetics, Centro Hospitalar Universitário São João, Porto, Portugal; 5 Institute for Research and Innovation in Health, Universidade do Porto, Porto, Portugal

**Keywords:** otosclerosis, stapes surgery, controlled hypotension

## Abstract

**Introduction:**

Stapes surgery (SS) benefits from adequate anesthetic bleeding control due to its microscopic nature and greater difficulty in hemostasis. Adequate hypotension during intervention is considered critical to achieving this goal.

**Objective:**

To evaluate the role of controlled hypotension (CH) on surgical outcomes in SS.

**Methods:**

We conducted a retrospective observational study in a tertiary academic center with adults submitted to SS from January 2017 to October 2022. Hypotension was considered controlled if the mean intraoperative arterial pressure was between 50 and 65 mmHg throughout the procedure. Patients were divided into CH and non-CH groups. Demographic data, medical history, anesthetic and surgical reports, perioperative complications, and audiometry results were studied.

**Results:**

A total of 99 SS procedures were included. Of those, 23 met the criteria for CH. The non-CH group presented a nonsignificant longer operative time (71.4 ± 21.3 versus 83 ± 27.4 minutes; *p* = 0.065). No statistical difference was observed in complication rates related to the procedure (39.1 vs. 48.7%; *p* = 0.421). In terms of audiometric data, both groups showed comparable pre- and postoperative pure tone average (PTA) results (preoperative: 55.4 ± 11.3 versus 53.1 ± 16.2dB; *p* = 0.525; postoperative: 31.6 ± 10.2 versus 33 ± 17dB; *p* = 0.722). The postoperative average air-bone gap (ABG) was 9.3 ± 8.3 versus 10.1 ± 9dB; *p* = 0.709, while closure of the ABG was 24 ± 13.1 versus 19.7 ± 17dB; *p* = 0.287.

**Conclusion:**

Controlled hypotension in SS does not appear to impact the audiometric outcomes or complication rates, despite having a potential role in the surgery duration.

## Introduction

Otologic surgery, including stapes surgery (SS), requires optimal visibility of the surgical field. Due to image magnification by the microscope or endoscope, even residual bleeding can easily obstruct the operative field, increasing risks associated with inner ear suction and loss of anatomic landmarks. Furthermore, unlike other procedures, vascular supply of the middle ear does not allow other forms of hemostasis, such as clamping.

Multiple approaches, such as hyperventilation, patient positioning, and intraoperative controlled hypotension (CH), have been tried to improve this condition. 1 This concept of CH, which aims to provide an optimal surgical field, was established several decades ago, 2 and seeks to achieve a systolic blood pressure of 80 to 90 mmHg, to reduce the mean arterial pressure (MAP) to between 50 and 65 mmHg or to reduce it by 30%. 1 3 This simultaneously optimizes the surgical field and enhances professional satisfaction in procedures such as tympanoplasty and sinonasal endoscopic surgery. 3 4 5 6

However, the effect on postoperative outcomes in SS is uncertain. The present study evaluates the impact of CH on surgical and postoperative outcomes in SS.

## Methods

### Study Design

We conducted a retrospective observational study in a tertiary hospital center with patients aged more than 18 years who had SS, from January 2017 to October 2022. Those who underwent revision SS, a simultaneous surgical procedure, or did not present complete audiometric and anesthetic data were excluded. Patients were divided into two groups according to the intraoperative MAP (IMAP) values: a group between 50 and 65 mmHg throughout the whole surgery, and a second group above 65 mmHg.

### Data Collection

The data collected included age, sex, comorbidities, tympanogram, acoustic reflexes, and pre- and postoperative air and bone thresholds. Pure tone average (PTA) and air-bone gap (ABG) were registered. We classified surgical techniques and applied materials, such as type and size of the prosthesis, as well as oval window sealing. Moreover, data on operative time was registered. Both intra- (such as nerve damage and perilymphatic gusher) and postoperative (such as vertigo, tympanic perforation, hypoacusis, tinnitus, nerve impairment, and prosthesis dislocation) complications were assessed.

### Anesthesia

During surgery, heart rate (HR) and MAP were recorded. Both were measured by the automated oscillographic method before the operation and at intervals of 10 or 15 minutes during the surgery (Datex-Ohmeda). Anesthetic induction was performed by 2 to 2.5 mg/kg propofol, 1 mcg/kg bolus of remifentanil, and 0.6 mg/kg of rocuronium in every patient. After endotracheal intubation, anesthesia was maintained with propofol, sevoflurane, and remifentanil. The patient's head was extended (15–30°) and rotated away from the surgeon according to the ear. The skin of the ear canal was infiltrated with 2% lidocaine, with a 1:100.000 adrenaline solution.

### Data Analysis

The statistical analysis was conducted with IBM SPSS Statistics for Windows (IBM Corp.) software, version 27.0. The continuous variables were expressed as mean and standard deviation (SD) or median and interquartile range (IQR) values, and the categorical variables, as numbers and percentages. The student's *t* -test, the Mann-Whitney U test, Chi-squared test, and the Fisher's exact test were used according to variable type. Statistical significance was set as *p* < 0.05.

## Results

A total of 206 patients underwent SS during the period evaluated. A total of 53 patients who were submitted to revision surgeries or associated with other procedures were excluded. An additional 54 patients were removed due to insufficient data. A final total of 99 were included in the study, of which 23 satisfied the CH criteria.

Patients' characteristics are presented in Table 1 . Both groups did not differ in terms of age (45.2 ± 9 versus 44.5 ± 10.3 years; *p* = 0.779) or on sex ratios (65.2 versus 77.6%. *p* = 0.230). Preoperatively, the MAP values were not significantly different between the groups (92 ± 19.9 versus. 96.9 ± 18.1 mmHg; *p* = 0.284), as shown in Table 2 .

**Table 1:** Characteristics of the study sample

	50–65 mmHg (n = 23)	> 65 mmHg (n = 76)	*p* -value
**Mean age in yearŝ**	45.2 ± 9	44.5 ± 10.3	0.779
**Female sex: n (%)**	15 (65.2)	59 (77.6)	0.23
**Medical history: n (%)**			
Hypertension	1 (4.3)	7 (9.2)	0.677
Diabetes mellitus	0 (0)	2 (2.6)	1
Smoking	2 (8.7)	4 (5.3)	0.621
Cardiovascular events	0 (0)	2 (2.6)	1
**Bilateral disease: n (%)**	11 (47.8)	39 (51.3)	0.769
**Right ear: n (%)**	11 (47.8)	41 (53.9)	0.606
**Follow-up in months: median (range)**	9 (11)	12 (14)	0.264

**Table 2:** Preoperative evaluation of the study sample

	50–65 mmHg (n = 23)	> 65 mmHg (n = 76)	*p* -value
**Mean preoperative arterial pressure (mmHg)**	92 ± 19.9	96.9 ± 18.1	0.284
**Tympanogram: n (%)**			0.096
A	12 (52.2)	48 (73.8)	
As	11 (47.8)	16 (24.6)	
C	0 (0)	1 (1.5)	
**Acoustic reflex: n (%)**			0.361
Absent	21 (91.3)	46 (75.4)	
Present	0 (0)	1 (1.6)	
On/Off	2 (8.7)	14 (23)	

Table 3 portrays surgical parameters. The most common procedure in both groups was stapedotomy (CH: 78.3%; Non-CH: 81.6%; *p* = 0.765). A statistically significant difference was observed in the sealing method between the 2 groups ( *p* < 0.001), specifically in the use of hemostatic gelatin sponge (CH: 13%; Non-CH: 35.5%; *p* = 0.04) and subcutaneous tissue (CH: 30.4%; Non-CH: 1.3%; *p* < 0.001). In the CH group, there were less sealing procedures (34.8%), while in the second group, sealing with perichondrium (36.8%) was favored. Intraoperative mean heart rate was similar between the 2 groups (CH: 66 ± 13.6 beats per minute [bpm]; Non-CH: 68 ± 10.4 bpm; *p* = 0.451).

**Table 3:** Surgical parameters of the study sample

	50–65 mmHg (n = 23)	> 65 mmHg (n = 76)	*p* -value
**Stapedotomy: n (%)**	18 (78.3)	62 (81.6)	0.765
**Piston length (mm): n (%)**			0.136
4	4 (19)	14 (18.7)	
4.25	4 (19)	4 (5.3)	
4.5	13 (62)	57 (76)	
**Piston diameter (mm): n (%)**			0.44
0.4	3 (15.8)	5 (7)	
0.6	15 (78.9)	57 (80.3)	
0.8	1 (5.3)	9 (12.7)	
**Piston material: n (%)**			0.080
Teflon	20 (87)	74 (97.4)	
Titanium	3 (13)	2 (2.6)	
**Sealing material: n (%)**			0.001
Absent	8 (34.8)	20 (26.3)	0.43
Hemostatic gelatin sponge	3 (13)	27 (35.5)	0.04
Perichondrium	4 (17.4)	28 (36.8)	0.081
Subcutaneous tissue	7 (30.4)	1 (1.3)	< 0.001
Blood clot	1 (4.3)	0 (0)	0.232
**Stapedotomy opening: n (%)**			1
Not otherwise specified	15 (65.2)	50 (65.8)	
Manual perforator	7 (30.4)	22 (28.9)	
Microdrill	1 (4.3)	4 (5.3)	
**Canalplasty: n (%)**			0.158
Not performed	7 (30.4)	21 (27.6)	
Curettage	13 (56.5)	30 (39.5)	
Microdrill	3 (13)	25 (32.9)	
**Mean heart rate (beats per minute)**	66 ± 13.6	68 ± 10.4	0.451

The non-CH surgeries showed a nonstatistically significant trend towards longer duration (CH: 71.4 ± 21.3 minutes; Non-CH: 83 ± 27.4 minutes; *p* = 0.065) as detailed in Table 4 . No differences in complication rates were observed (CH: 39.1%; Non-CH: 48.7%; *p* = 0.421). Concerning audiometric data, both groups presented similar mean preoperative PTA (CH: 55.4 ± 11.3 dB; non-CH: 53.1 ± 16.2 dB; *p* = 0.525 and mean preoperative ABG values (CH: 28.1 ± 8.2 dB; non-CH: 28.5 ± 9.1 dB; *p* = 0.859).

**Table 4:** Audiological outcomes, operative time, and complications

	50–65 mmHg (n = 23)	> 65 mmHg (n = 76)	*p* -value
**Mean operative time** in minutes	71.4 ± 21.3	83 ± 27.4	0.065
**Complications: n (%)**	9 (39.1)	37 (48.7)	0.525
**Preoperative period**			
Mean PTA	55.4 ± 11.3	53.1 ± 16.2	0.525
Mean ABG	28.1 ± 8.2	28.5 ± 9.1	0.859
**Postoperative period**			
Mean PTA (dB)	31.6 ± 10.2	33 ± 13.1	0.722
Mean ABG (dB)	9.3 ± 8.3	10.1 ± 9	0.709
**ΔABG** (dB)	24 ± 13.1	19.7 ± 17	0.287

**Abbreviations:** ΔABG, closure of the air-bone gap; ABG, air-bone gap; PTA, pure tone average.

Neither the mean postoperative PTA values (CH: 31.6 ± 10.2 dB; Non-CH: 33 ± 17 dB; *p* = 0.722) nor the mean postoperative ABG values (CH: 9.3 ± 8.3 dB; Non-CH: 10.1 ± 9 dB; *p* = 0.709) nor the mean ABG closure (CH: 24 ± 13.1 dB; Non-CH: 19.7 ± 17 dB; *p* = 0.287) showed significant changes.

## Discussion

To the best of our knowledge, this is the first paper to address the role of blood pressure control in the outcome of SS. The surgery is centered around the middle ear, addressing the stapes and oval window, while tympanoplasty focuses on the tympanic membrane, making SS more dependent on proper visualization of the middle ear. In our work, the non-CH surgeries were longer despite showing no statistically significant difference. Yet, other relevant factors should additionally be considered, namely surgeons' expertise, anatomical complexity, or the disease's specificities.

In the present study, both groups had similar audiometric results as well as complication rates. Despite adequate visibility of the operation field, as reported in previous studies, 7 8 9 this advantage may have limited impact on the outcome. The major purpose of CH, namely in orthopedic and maxillofacial surgery, is to reduce blood loss and, consequently, the need for transfusions. 10 11 Praveen et al. demonstrated that, in orthognathic surgery, hypotensive patients had less blood loss compared to normotensive patients. 12 However, regarding otological surgery, this benefit is negligible, and the use of this technique is supported by its association with better surgical conditions.

Controlled hypotension is based on the reduction of vascular resistance, to reduce hemorrhage in the surgical field. However, there are other phenomena that optimize surgical visibility and are not specifically owed to hypotension. Optimal cardiac output may play a relevant role.

Furthermore, heart rate can be fundamental in achieving ideal conditions. 13 In the present study, this value was similar in both groups, limiting an appropriate evaluation of this parameter. Pharmaceutical agents may also be relevant to the proposed objectives. The use of remifentanil to control hypotension has been proven effective in adults and children. 7 8 9 Regarding tympanoplasty, several studies by Degoute et al. have demonstrated its effectiveness in maintaining hypotension and improving surgeon satisfaction. 7 8 However, it appears that other substances can accomplish the same goal without affecting blood pressure. A study by Kosucu et al., 14 which evaluated the use of dexmedetomidine in tympanoplasty, revealed it provided an adequate operating field without the need for simultaneous hypotension.

Furthermore, CH can, paradoxically, also be an unhelpful technique, increasing bleeding. 6 14 It is recognized that the reduction of arterial hypotension may trigger local vasodilation. Moreover, hemorrhage in the operating field can be the result of a reflex tachycardia, which is known to happen under these conditions. One study showed the benefits of sodium nitroprusside in the surgical field only at very low blood pressure values. 6 Thus, by achieving optimal surgical conditions, patients may be exposed to the adverse effects of this technique.

Another important aspect is the technical complexity and health risks inherent in achieving this target blood pressure range. Patients with ischemia and hypertension face greater risks to attain a sustainable CH. Under these conditions, it is important to consider the risk of inadequate perfusion of vital organs, such as the brain and heart. 1 15 However, this concern may be overestimated, and the medical impact of CH appears to be modest. 11 16

The present study has several limitations. Despite evaluating multiple parameters, it was not possible to classify some variables. Evaluating surgeon's subjective satisfaction in the operative field could serve as a bridge in understanding the relationship between blood pressure and postoperative results, presenting a compelling topic for future research.

The surgical procedures were performed by several surgeons. However, apart from the sealing method of choice, the surgical techniques were similar. Another notable limitation is the small sample size. This factor resulted from the extensive collection of operational and follow-up parameters. Finally, indirect blood pressure measurement, while less accurate than direct, remains a useful and practical method. 17

## Conclusion

Controlled hypotension in stapes surgery did not impact the audiometric outcomes or complication rates. However, it may have a potential role in surgery duration. These findings highlight the need to reconsider routine use of controlled hypotension in stapes surgery.
